# Site-Directed Immobilization of *Pseudomonas fluorescens* Lipase Based on SnoopCatcher/SnoopTag System for Biodiesel Production

**DOI:** 10.3390/ijms26115385

**Published:** 2025-06-04

**Authors:** Baoyuan Zhang, Chenxi Zhao, Liangyu Zhao, Fenghuan Wang, Sai Wen

**Affiliations:** 1 Key Laboratory of Geriatric Nutrition and Health, Ministry of Education, Beijing Technology and Business University, Beijing 100048, China; 2 School of Light Industry Science and Engineering, Beijing Technology and Business University, Beijing 100048, China

**Keywords:** SnoopCatcher/SnoopTag system, directed immobilization, lipase, magnetic carrier

## Abstract

The site-directed immobilization of enzymes has demonstrated significant potential in industrial applications due to its ability to minimize enzyme heterogeneity and maximize retained activity. However, existing approaches often require the introduction of unnatural amino acids or excessive specific ligase to achieve this goal. In this study, a self-catalyzed protein capture system (i.e., the SnoopCatcher/SnoopTag pair) was utilized for the directed immobilization of lipase on magnetic carriers. By tagging the *Pseudomonas fluorescens* lipase (PFL) with a SnoopTag at the C-terminal, the fused lipase PFL-SnoopTag (PSNT) readily conjugated with the SnoopCatcher partner via a spontaneously formed isopeptide bond between them. Novel magnetic particles functionalized by SnoopCatcher proteins were prepared using a co-precipitation method, achieving a loading capacity of around 0.8 mg/g carrier for the SnoopCatcher. This functionalized magnetic carrier enabled the site-directed immobilization of lipase PSNT at 81.4% efficiency, while the enzyme loading capacity reached 3.04 mg/g carriers. To further assess the practical performance of site-directed immobilized lipases, they were applied in biodiesel production and achieved a yield of 88.5%. Our results demonstrate a universal platform for the site-directed immobilization of enzymes with high performance, which offers significant advantages, e.g., single-step purification and catalyst-free immobilization of engineered enzymes, as well as easy recovery, highlighting its potential for industrial applications.

## 1. Introduction

Enzyme immobilization technology aims to improve the catalytic performance and operational stability of a given enzyme under unfavorable conditions and facilitate the recovery of that enzyme by physically or chemically binding the free enzyme to a specific carrier [1]. Unfortunately, the traditional immobilization method randomly reacts the amino acid side chain of the enzyme with the functional groups on the immobilization matrix, which often changes the conformation of the enzyme or hinders the binding of the substrate to the reaction site of the enzyme, thus reducing catalytic activity [2]. Site-specific enzyme immobilization can attach enzymes to carriers via a single, predefined site, so that the enzyme can adopt an ordered orientation, leaving the active site free to maintain maximum activity. Traditionally, there are four main methods for the site-specific immobilization of enzymes [3]. First, the cysteine-mediated covalent binding of enzymes to a carrier through disulfide bonds has been extensively studied; however, introducing cysteine into the enzyme structure usually alters the protein conformation and reduces catalytic activity [4]. The second strategy is by click chemical reaction, which mostly involves the amino-alkynyl click reaction for enzyme immobilization. Although the amino-alkynyl reaction can take place spontaneously, the reaction efficiency is low and a cross-linking agent needs to be added, resulting in poor mechanical stability of the carrier [5]. There are also the affinity labeling method [6] and enzyme-catalyzed method [7]. Affinity-labeled carriers are often prohibitively expensive, and the suboptimal binding stability between the affinity tag and its ligand may compromise the reusability of the immobilized enzymes. Enzyme-catalyzed immobilization methods require the addition of exogenous bond-forming enzymes (e.g., the phosphopantetheinyl transferase Sfp) to achieve effective conjugation between the target enzyme and the carrier matrix. Meanwhile, such methods typically involve complex, multi-step procedures, and the heterogeneous spatial distribution of the enzymes on carriers frequently diminishes the catalytic efficiency. The inherent limitations of these methods pose significant challenges in practical applications [8].

In the past decade, self-catalyzed biomolecular click reaction systems have been discovered and have received increasing attention [9]. These systems originated from several bacterial surface protein domains and can spontaneously form an isopeptide bond between the reactive lysine and aspartic acid (or asparagine) residues residing separately in the Catcher and Tag partners. Compared with traditional click chemistry, genetically encoded Catcher/Tag systems are characterized by mild reaction conditions, orthogonal reactivity, and the absence of a need for catalysts or the introduction of unnatural amino acids, thereby enabling the highly specific conjugation of proteins both in vivo and in vitro. Among these systems, the Snoop system is a recently reported system derived from the adhesin of *Streptococcus pneumoniae*, whereby the isopeptide bond can spontaneously form between Lys742 (SnoopTag) and Asn854 (SnoopCatcher) in its D4Ig-like domain [10]. This system has proved to be efficient under a wide range of temperatures and redox conditions and even with various detergents. Unlike the widely used Spy system, for which the optimal reaction pH range is 5–6, the Snoop system expands the reaction pH to more than 9, giving rise to the possibility of developing orthogonal reactions between different Catcher/Tag pairs. At present, the SnoopCatcher/SnoopTag pair has been used alone or coupled with other Catcher/Tag pair(s) for antibody polymerization [10], monomolecular tethering [11], protein purification [12], and 3D bionanomaterial assembly [13], but its application in enzyme immobilization remains underexplored. Therefore, it is of great interest to explore the potential of the SnoopCatcher/SnoopTag system for the site-directed immobilization of tagged enzymes on modified carriers for industrial applications.

**Figure 1:**
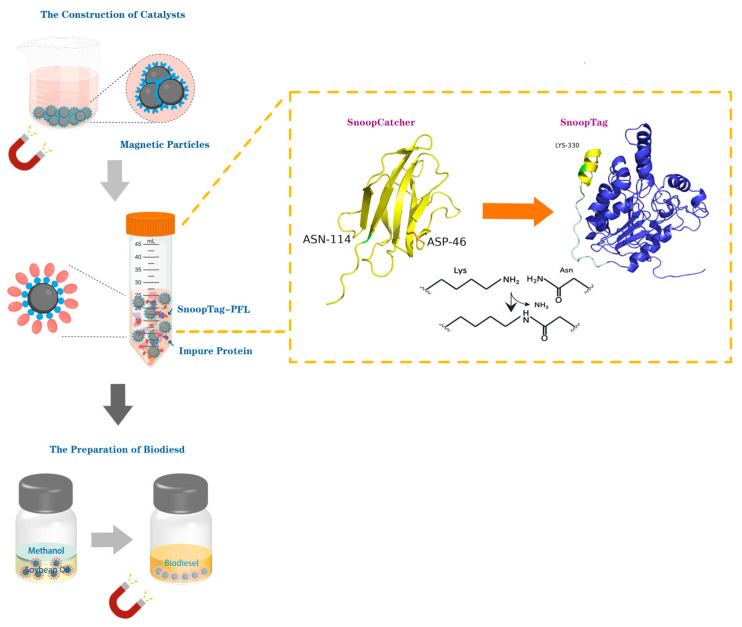
Schematic illustration of the preparation of SnoopCatcher-modified magnetic particles incorporating site-directed immobilized lipases for biodiesel production.

The most significant advantage of a magnetic material as a carrier matrix is that it can be easily separated under an external magnetic field. Even in non-aqueous phase reaction solutions, this also enhances the operational practicality, which provides a solid support for the immobilization of enzymes [14]. In this study, a supporting magnetic carrier modified by SnoopCatcher protein was prepared by a co-precipitation method. Meanwhile, a lipase from *Pseudomonas fluorescens* (PFL) linked with the SnoopTag through a linker peptide, enabling efficient capture by SnoopCatcher-modified magnetic particles, was constructed and was shown to maintain both enzymatic and ligation activities. We further demonstrated the utility of this immobilized lipase for the production of biodiesel. In this work, we demonstrate the preparation of Catcher-modified magnetic carriers for the site-directed immobilization of enzymes via a one-step synthesis and their use in specific enzyme binding from cell lysates (Figure 1).

## 2. Results

### 2.1. Construction and Expression of Snoopcatcher Protein and Snooptag Tagged Lipase

The SnoopTag/SnoopCatcher pair was derived from the D4 Ig-like domain of the adhesin RrgA from *S. pneumoniae*, which naturally forms an intramolecular isopeptide bond to assist the colonization of the bacteria. The SnoopTag peptide is a small peptide of 12 amino acids with an isopeptide bond-forming Lys residue, while its protein partner SnoopCatcher has 115 amino acids, containing the reactive Asn residue and a catalytic Glu residue. In this research, the protein SnoopCatcher with a 6 × His Tag at its C-terminus was designed to facilitate protein purification by affinity chromatography. As for the recombinant lipase PFL, it was fused to the SnoopTag peptide via a rigid PT Linker (PTPPTTPTPPTTPTPTP) to decrease the likelihood of interaction between SnoopTag and PFL lipase, which may have affected their functionality.

**Figure 2:**
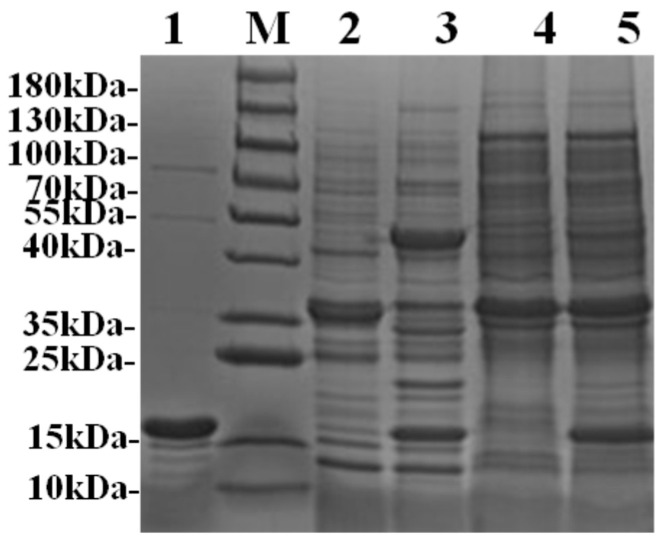
The soluble protein samples of SNC, PSNT, and SNTP were analyzed by SDS-PAGE and examined for their reactivity. (M: protein marker; 1: SNC protein; 2: inclusion bodies of PSNT dissolved in sodium deoxycholate; 3: mixture of SNC and PSNT proteins; 4: inclusion bodies of SNTP dissolved in sodium deoxycholate; 5: mixture of SNC and SNTP proteins).

The SnoopCatcher (SNC), PFL-SnoopTag (PSNT), and SnoopTag-PFL (SNTP) genes were constructed, cloned into expression vectors, and then transformed into *E. coli* BL21(DE3) cells. The corresponding positive clones were induced by IPTG at 18 °C for intracellular expression of the target proteins. Our result showed that the SnoopCatcher protein SNC was efficiently expressed as soluble proteins, whereas the tagged lipases were mainly expressed as inclusion bodies. To increase the solubility of the inclusion bodies, the precipitates were solubilized overnight in PBS containing 0.2% (*w*/*v*) sodium deoxycholate, and the supernatant was collected for assay. All three soluble crude protein samples were then verified by SDS-PAGE, which showed specific bands with the theoretical molecular weights of SNC (15.4 kDa) and PSNT/SNTP (36.2 kDa) (Figure 2). Quantitative analysis showed that the recombinant proteins SNC, PSNT, and SNTP were expressed at yields of 90.5 mg/L, 19.7 mg/L, and 9.52 mg/L, respectively.

Due to the well-characterized high binding specificity between SnoopCatcher and SnoopTag, we evaluated the conjugation efficiency between SNC and PSNT/SNTP by directly incubating crude protein samples with excess SNC. As shown in Figure 2, SDS-PAGE analysis revealed a distinct band migrating at a molecular weight corresponding to the sum of that of SNC and PSNT, indicating the formation of an irreversible covalent bond between the two proteins. This result demonstrates the feasibility of utilizing this interaction system for enzyme directed immobilization in turbid biological matrices (e.g., cell lysates) without prior purification, representing a significant advantage over conventional immobilization strategies that require labor-intensive purification steps.

**Figure 3:**
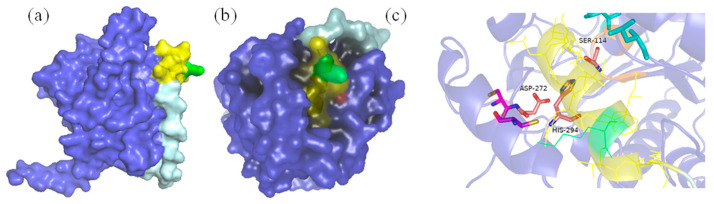
Simulated structures of PSNT and SNTP for comparison (blue for PFL, cyan for PTLinker, yellow for SnoopTag, and green for reactive Lys residue). (**a**): PSNT surface structure; (**b**): SNTP surface structure; (**c**): The localized structure of SNTP’s catalytic center (pink for lipase catalytic triad of Ser-His-Asp).

By comparison, no specific conjugation was observed between SNC and SNTP under the same conditions. To investigate the molecular basis of this failure, we performed structural comparisons between PSNT and SNTP (Figure 3). Molecular modeling revealed that the N-terminal fusion of SnoopTag resulted in its unexpected insertion into the catalytic pocket of PFL lipase. The primary helix of SnoopTag, which normally projects outward for SnoopCatcher binding, directly occupied the enzyme’s active site, creating steric clashes for the SnoopTag’s binding interface and PFL’s substrate-access channel. This structural conflict explains why N-terminal fusions failed to bind SnoopCatcher, whereas C-terminal fusion, in which the α-helical bundle of SnoopTag remained fully solvent-exposed, maintained high conjugation efficiency. Based on these findings, the C-terminal fusion, i.e., PSNT, was selected for subsequent experiments and characterization.

### 2.2. Preparation of SnoopCatcher Modified Magnetic Particles

Magnetic Fe_3_O_4_ (magnetite) particles can be synthesized through methods like co-precipitation, where iron salts are precipitated in the presence of a base, and hydrothermal or solvothermal methods, where the reaction occurs under high pressure and temperature in a controlled solvent environment. Other techniques include sol-gel, microemulsion, and mechanical grinding methods. Among these, the co-precipitation method is the most widely adopted approach due to its straightforward protocol, rapid synthesis, and cost-effectiveness. Therefore, SnoopCatcher-functionalized magnetic particles were prepared via the co-precipitation approach. Specifically, Fe^2+^ and Fe^3+^ ions in a 1:2 molar ratio were dissolved in 0.5 mol/L acetic acid as the reaction medium, followed by the addition of a certain amount of SNC proteins. Subsequent alkalization with ammonia under continuous mechanical agitation yielded monodisperse magnetic particles, which were easily collected using a magnet.

A quantitative assessment of the SNC protein concentrations in the pre-immobilization and post-immobilization supernatants revealed an inverse correlation between protein input and immobilization yield, with saturation observed at a maximal loading capacity of 0.8 mg SNC per 15 mL reaction system (Table 1). Accordingly, an optimal SnoopCatcher input of 1.0 mg/15 mL was adopted to balance cost-efficiency with preserved catalytic activity, a parameter subsequently standardized for SNC-functionalized magnetic particle preparation.

### 2.3. Immobilization of Pfl on Modified Magnetic Carriers

In a previous study, the reaction of SnoopTag (fused with MBP) with excess SnoopCatcher to a high degree of completion took almost an hour [15]. Therefore, we speculated that a longer time course of directed immobilization of tagged lipase PFL may be required due to the reduced flexibility of SnoopCatcher proteins fixing on magnetic carriers. To determine the optimal reaction time for immobilization, 3.7 mg of PSNT dissolved in 10 mL Tris-HCL buffer (20 mM) was mixed with 1g of SNC modified magnetic particles, and the immobilization efficiency was calculated at indicated times. As shown in Table 2, the immobilization efficiency reached 81.4% after 8 h of incubation and almost stagnated as time progressed. Therefore, 8 h was selected as the incubation time for PFL immobilization. Since the incubation conditions were not as alkaline as required for the reaction between hydroxyl groups (on the magnetic particle) and carboxyl groups (on the protein), the magnetic particles did not directly covalently conjugate with PSNT [16]. Instead, covalent binding was mediated by the SNC immobilized on magnetic particle surface. Under this condition, the apparent lipase activity of freeze-dried immobilized enzyme powder was around 142 U/mg carrier by the *p*-nitrophenol method.

**Figure 4:**
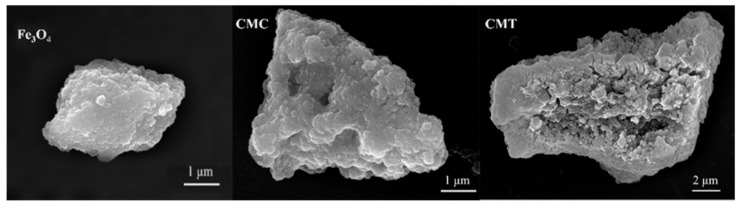
SEM images of the surface morphology of unmodified magnetic particles (Fe_3_O_4_), Fe_3_O_4_ particles modified by SNC (CMC), and CMC particles with immobilized PSNT proteins (CMT).

The morphology of the synthesized magnetic carriers was characterized through scanning electron microscopy (SEM). The SEM images revealed that the unmodified magnetic particles had a rough and uniform surface. In contrast, after sequential modifications with SNC and PSNT proteins, the surface morphologies of the particles exhibited substantial changes, including increased roughness and the formation of large cavities (Figure 4). These findings suggest that the incorporation of SNC proteins via the co-precipitation method significantly altered the structural characteristics of the magnetic particles. To understand the molecular mechanisms underlying these morphological changes, FIRT analysis was then conducted.

**Figure 5:**
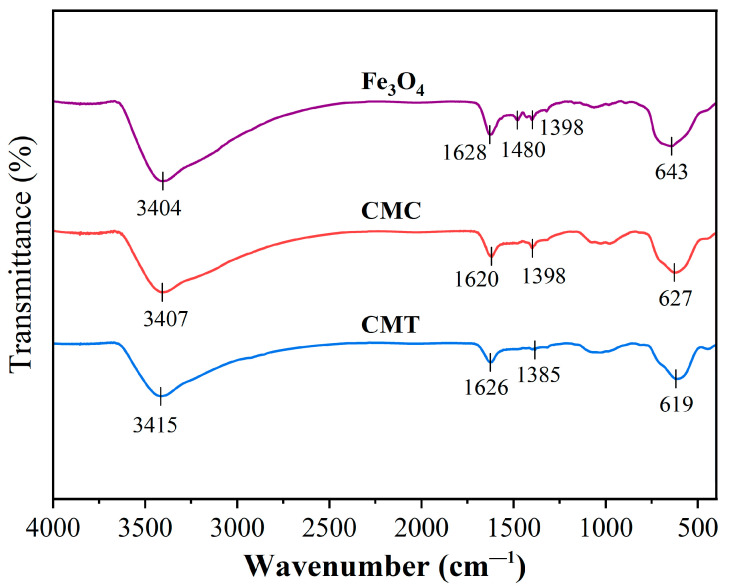
Fourier transform infrared spectra of unmodified magnetic particles (Fe_3_O_4_), Fe_3_O_4_ particles modified by SNC (CMC), and CMC particles with immobilized PSNT proteins (CMT).

FTIR analysis (Figure 5) revealed progressive surface modifications across functionalized particles. Native Fe_3_O_4_ displayed characteristic O-H stretching (3404 cm^−1^), H-O-H bending (1628 cm^−1^), and Fe-O lattice vibrations (643 cm^−1^), alongside carbonate impurities (1480/1398 cm^−1^) from ambient CO_2_ adsorption. Upon SNC immobilization (sample CMC), attenuated O-H intensity (32% reduction at 3407 cm^−1^) and redshifted carboxylate vibrations (1620 cm^−1^, Δν = −8 cm^−1^) confirmed covalent conjugation between the carboxyl groups of SNC and hydroxyls of Fe_3_O_4_ via condensation reaction. Further functionalization with PSNT (sample CMT) exacerbated the O-H/N-H signal attenuation (55% intensity loss at 3415 cm^−1^), consistent with hydrogen bonding between PSNT (-NH_2_) and SNC (-NH_3_^+^), while amide I overlap (1626 cm^−1^) and Fe-O peak displacement (619 cm^−1^) suggested protein structural reorganization and loading-induced lattice distortion. Collectively, these spectral trends demonstrated (1) SNC anchoring via carboxyl-hydroxyl covalent linkage, (2) PSNT assembly through polar amino acid interactions, and (3) progressive functional group shielding by multiple protein adlayers.

**Figure 6:**
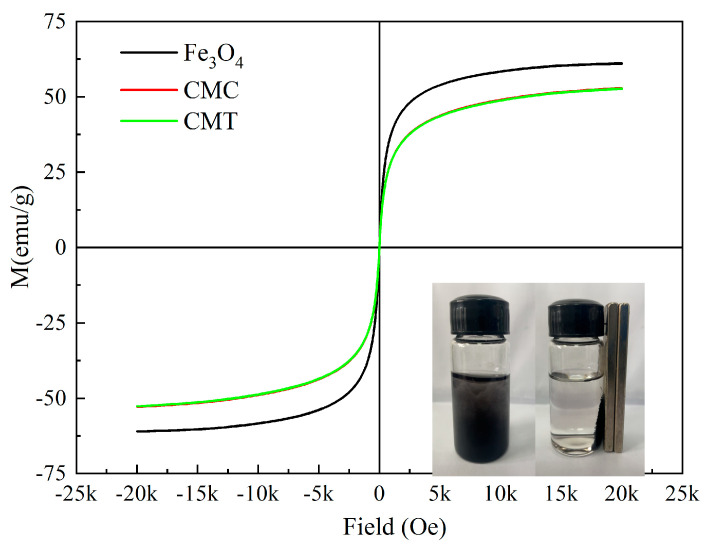
Hysteresis loop of unmodified magnetic particles (Fe_3_O_4_), Fe_3_O_4_ particles modified by SNC (CMC), and CMC particles with immobilized PSNT proteins (CMT).

To further investigate the potential influence of SNC and PSNT adlayers on the magnetic properties of the carriers, the magnetic moments of the particles were analyzed using a vibrating sample magnetometer (VSM). As illustrated in Figure 6, the saturation magnetization of the Fe_3_O_4_ samples exhibited a slight reduction after modification with SNC and PSNT proteins. Notably, the residual magnetization and magnetic coercivity of all three types of particles were measured to be zero, confirming that their superparamagnetic behavior had been retained. This superparamagnetic property ensured that the particles could be efficiently separated and recovered under an external magnetic field (Figure 6).

### 2.4. Biodiesel Production Catalyzed by Site-Directed Immobilized Lipases on Magnetic Carriers

**Figure 7:**
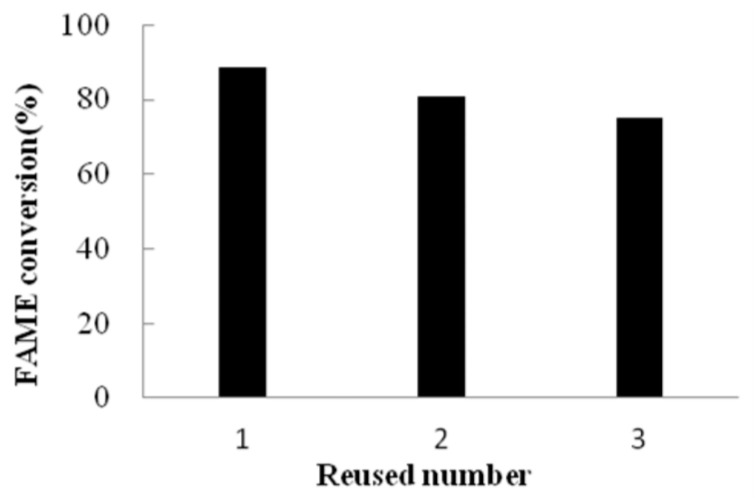
The reusability of the immobilized lipase on magnetic carriers.

Biodiesel, a pivotal renewable energy source comprising long-chain fatty acid alkyl esters synthesized from renewable feedstocks such as vegetable oils, animal fats, and recycled greases, exhibits combustion properties like those of conventional petrodiesel while offering the significant environmental advantages of net carbon dioxide emission reductions and enhanced fuel combustion efficiency [17]. Lipase-catalyzed transesterification has emerged as a sustainable alternative to conventional alkaline processes, circumventing drawbacks such as saponification and excessive wastewater generation. In the present study, the transesterification efficacy of immobilized lipase PFL on magnetic carriers was evaluated under optimized conditions: 200 mg of magnetic carriers with immobilized PFL, a molar substrate ratio (oil to methanol) of 1:4, a controlled water content of 2% (*w*/*w*) (Aw = 0.72), and a reaction duration of 72 h at 45 °C with continuous agitation (200 rpm). Quantitative gas chromatography (GC) analysis was conducted using methyl salicylate as the internal standard. The calibration was performed with a mixture of three methyl esters (methyl palmitate, methyl oleate, and methyl linoleate), whose formation in the biodiesel synthesis process was confirmed. The final conversion rate of fatty acid methyl esters (FAME) reached 88.5%. Following the retrieval of PFL-immobilized magnetic particles and their subsequent application to two successive catalytic cycles, the FAME conversion rate exhibited a slight decline while remaining above 75% (Figure 7). A comparative analysis revealed that an equivalent amount of free lipase PFL failed to catalyze biodiesel production, mainly attributed to the severe aggregation of PFL powder in the reaction system. This operational stability assay demonstrated robust reusability of the prepared catalysts incorporating site-directed immobilized PFL. Moreover, the magnetic matrix-supported catalysts facilitated rapid and efficient enzyme recovery from the biphasic reaction medium via external magnetic separation, underscoring their practical utility in industrial processes.

## 3. Discussion

Enzyme immobilization on magnetic particle carriers offers dual advantages of enhanced enzymatic stability and convenient recovery. Nevertheless, traditional immobilization approaches for engineered enzymes derived from crude samples are equally inefficient, due to their multi-step procedures for protein purification and the binding of enzymes. In this study, the SnoopCatcher/SnoopTag system was employed to achieve the specific and directional conjugation of enzymes through self-catalyzed covalent bond formation. Our protocol involved (1) surface-functionalization of carriers with SnoopCatcher domains, and (2) genetic fusion of SnoopTag to target lipases, enabling site-specific immobilization via molecular recognition pairing. This immobilization method was shown to be highly specific and efficient, enabling the one-step purification and immobilization of enzymes from cell lysates, which would significantly lower production costs and reduce environmental impact. Notably, a comparable approach employing the SpyCatcher/SpyTag system was recently reported for multi-enzyme co-immobilization on Fe_3_O_4_@SiO_2_-Br nanoparticles. However, the protocol required the solid-phase synthesis of Tag peptides through multi-stage chemical reactions, whereas our method utilizes recombinantly expressed SnoopCatcher proteins produced via *E. coli* expression systems with a high yield. Our biological production route not only simplifies carrier modification but also enhances conjugation efficiency, considering that the larger Catcher proteins with more carboxyl groups are more likely to undergo condensation reactions with the hydroxyl groups of Fe_3_O_4_ particles, compared to small peptides like Tags.

The SpyTag/SpyCatcher fusions have been validated across diverse fusion architectures, including fusion on the N- terminus or C-terminus, in an exposed linker or in loop regions, showing the outstanding positional flexibility and fusion tolerance of these system [18,19]. In contrast, our study presents the SnoopTag/SnoopCatcher system. The SnoopTag retained its reactivity when fused at the C-terminus of lipase PFL, while the N-terminal fusion resulted in conjugation failure due to steric occlusion of its interaction sites by PFL’s catalytic domain. This positional dependence suggested structural constraints imposed by specific target proteins—particularly those with surface regions that could compromise SnoopTag accessibility. These findings underscore the necessity of empirical optimization for fusion site selection in Tag/Catcher applications. When initial constructs exhibit suboptimal activity, switching the Tag to the opposite terminus may restore functionality, as conformational flexibility is critical for pairing between Tag and Catcher domains.

In this study, we established a universal enzyme immobilization platform via the SnoopCatcher/SnoopTag system. This platform, based on SnoopCatcher-modified magnetic particles, enabled the efficient capture of SnoopTag-labeled enzymes from crude cell lysates and reduced enzyme heterogeneity for improved performance. We demonstrated its effectiveness in the site-directed immobilization of lipase PFL and used it to catalyze biodiesel production in a heterogeneous system. Due to the genetic flexibility of SnoopTag in terms of its organization with other enzymes, this platform offers a versatile and broadly applicable approach for enzyme immobilization.

## 4. Materials and Methods

### 4.1. Materials

The structural genes of SnoopCatcher (GenBank: KU500646.1), SnoopTag (GenBank: KY978851.1) and PFL (ABA72315.1) were optimized according to the codon preference in *E. coli*, and the plasmids pUC57-SnoopCatcher and pUC57-PFL-PT Linker-SnoopTag were synthesized. *E. coli* Top10 competent cells and *E. coli* BL21(DE3) competent cells were purchased from Beijing Zhuangmeng Technology Co., Ltd. (Beijing, China). Plasmid pET28a were preserved in this lab.

### 4.2. Construction of pET28a-SnoopCatcher and pET28a-PFL-SnoopTag Plasmids

As shown in Table 3, primers were designed for the cloning of SnoopCatcher and PFL-SnoopTag genes with pUC57-SnoopCatcher and pUC57-PFL-PT Linker-SnoopTag plasmids as the PCR template, respectively.

The target genes were recovered by a gel purification kit and ligated with the TA vector by One step ZTOPO-Blunt/TA cloning kit, and then the cloning plasmids TA-SnoopCatcher and TA-PFL-SnoopTag were successfully constructed and verified by DNA sequencing. The target genes in the cloning vector and the expression plasmid pET28a were both double digested with restriction endonuclease *XhoI* and *NcoI*, before being ligated by T4 ligase and transformed into *E. coli* BL21(DE3) competent cells.

### 4.3. Expression of Fusion Proteins

The *E. coli* BL21(DE3) strain containing the target plasmid pET28a-SnoopCatcher or pET28a-PFL-PTlinker-SnoopCatcher was induced by IPTG to express the protein SnoopCatcher (SNC) or PFL-SnoopTag (PSNT) at 18 °C 220 rpm [20]. After induction, the cells were collected by centrifugation at 8000 rpm for 10 min at 4 °C. The bacteria were washed twice with 20 mM Tris-HCL buffer (pH 7.4), and the weight of the bacteria was suspended in the buffer. The bacterial body suspension was broken twice under the condition of a high-pressure homogenizer at 1500 bar, and the broken liquid was collected. After centrifugation at 8000 rpm for 10 min, the supernatant collected by SNC was the crude protein enzyme solution, and the inclusion body collected by PSNT was the target protein sample. The end of the SNC protein was designed with a 6 × His Tag, and the crude enzyme solution was subjected to Ni column affinity chromatography to remove the impurity protein before being desalted by molecular sieves to obtain the purified protein SNC. The PSNT inclusion body was dissolved using 0.2% sodium deoxycholate, and the supernatant was collected by centrifugation at 8000 rpm for 20 min to collect the crude protein enzyme solution.

In 96-well plates, 15 μL of purified protein sample with an appropriate concentration was added, followed by 285 μL of Coomassie brilliant blue dye solution. The wells were mixed evenly and placed at room temperature for 5–10 min. The absorbance at 595 nm was measured using a microplate reader and the readings were recorded. Standard curves were made using the light absorption data of the BSA protein samples [21].

### 4.4. Determination of Fusion Lipase Activity

The activity of PSNT was determined using the *p*-nitrophenol method with *p*-nitrophenyl palmitate (*p*-NPP) as the substrate. One unit of enzyme activity (U) was defined as the amount of enzyme required to produce 1.0 μmol *p*-nitrophenol per minute at 45 °C [22]. The calculation formula is as follows:

$$U=\frac{{∆OD}_{410}\times V}{t\times \varepsilon \times V_{enzyme}\times C_{enzyme}}$$

where ΔOD_410_ refers to the change value of OD_410_ in the reaction time, *V* represents the total reaction volume (mL), *ε* represents the extinction coefficient of nitrophenol, *t* represents the reaction time (min), *V*_enzyme_ represents the volume of enzyme added in the experiment (mL), and *C*_enzyme_ represents the concentration of enzyme (mg/mL).

**Figure 8:**
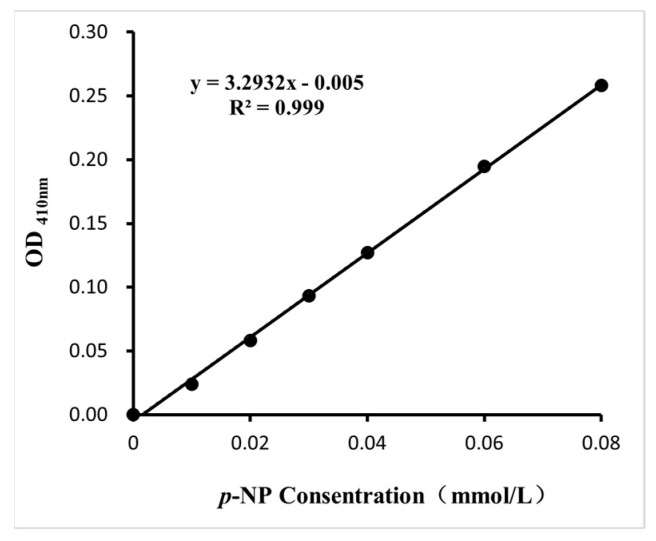
Standard curve of enzyme activity with *p*-NP as standard substrate.

With *p*-NP as standard substrate, the standard curve of lipase activity was obtained, as shown in Figure 8.

### 4.5. Verification on the Conjugation of Protein Pairs

The purified desalted SNC protein was fused with the PSNT fusion protein dissolved in sodium deoxycholate at a volume ratio of 1:1, pH 8.0, 25 °C, standing for 2 h. Protein binding was verified by SDS-PAGE protein electrophoresis. The unmixed protein components of the same concentration were used as negative controls.

### 4.6. Preparation of SNC Modified Magnetic Particles (CMC)

SNC modified magnetic particles (CMC) were prepared by the co-precipitation method. An appropriate amount of SNC protein was dissolved in 100 mL of 0.5 mol/L acetic acid solution. Subsequently, 18.10 g of FeCl_3_·3H_2_O and 9.30 g of FeSO_4_·7H_2_O were added to the solution and fully stirred until dissolved. Next, we added 50 mL of ammonia and mixed the solution. The reaction mixture was first stirred at room temperature for 1 h, followed by heating at 65 °C for an additional 30 min to complete the process [23].

After ripening, the reaction solution was cooled to 25 °C, and the black precipitate was separated by a magnet. Subsequently, the protein concentration in the reaction supernatant was detected, and the immobilization efficiency of the protein was calculated accordingly. To remove the excess ions and proteins in the precipitate, the precipitate was washed with water many times until the washing solution was neutral. Finally, CMC powder was obtained by low temperature vacuum drying. The immobilization efficiency of SNC was calculated as follows [24]:

$$\begin{array}{l} Protein\;immobilization\;efficiency\;(\%)\\ \;\;\;\;\;\;=\frac{Protein\;content\;before\;immobilization\left(mg\right)-Protein\;content\;after\;immobilization\left(mg\right)}{Total\;protein\;before\;immobilization\left(mg\right)} \end{array}$$

### 4.7. Immobilization of Recombinant Lipase Psnt

The recombinant protein PSNT was mixed with a certain amount of CMC and ligated at 4 °C for a certain time. The protein concentration and enzyme activity of the supernatant were detected, and the enzyme immobilization efficiency and enzyme activity recovery rate were calculated. The calculation formula of enzyme activity recovery rate was as follows [25]:

$$recovery\;of\;enzyme\;activity=\frac{Total\;activity\;of\;immobilized\;enzyme}{Total\;activity\;of\;free\;enzyme\;added}\times 100\%$$

After the reaction, the CMC was adsorbed by the magnet to remove the crude enzyme solution, the crude enzyme solution was washed three times with water, and the immobilized lipase powder (CMT) was obtained after low temperature vacuum drying.

### 4.8. Carrier Characterization

#### 4.8.1. SEM

The morphology of the carrier was observed by scanning electron microscopy (Zeiss 300, Jena, Germany). Before the test, the sample was dispersed in ethanol for 10 min, dispersed on the conductive adhesive, and tested at about 150 KV.

#### 4.8.2. VSM

The magnetization of the sample was measured at room temperature using a vibrating sample magnetometer from LakeShore, Westerville, OH, USA, with a scanning range of −20,000 Oe to 20,000 Oe [26].

### 4.9. Biodiesel Production Catalyzed by Immobilized Lipase

Reaction system: Immobilized lipase was used as biocatalyst for biodiesel. The transesterification reaction mixture was mixed with a certain amount of immobilized enzyme, 1.3 g soybean oil, and a certain amount of anhydrous methanol (the molar ratio of oil to methanol was 1:4). Methanol was added three times, once every 8 h, with a 2% water content at 45 °C, 200 r/min, for 72 h. After the reaction was complete, the enzyme was immobilized by magnetic separation at 12,000 r/min, and the supernatant was centrifuged for 10 min. The supernatant after centrifugation was analyzed by gas chromatography [27].

Water activity measurement: The water activity (Aw) of the biodiesel reaction system was measured using an AQUALAB 4TEV Water Activity Meter (METER Group, Inc., Pullman, WA, USA). Prior to testing, the instrument was calibrated using standard salt solutions. Samples were loaded into dedicated sample cups, and Aw values were recorded, with the average value calculated from triplicate measurements.

Detection of biodiesel by gas chromatography: The supernatant after centrifugation of 10 μL was mixed with 300 μL of internal standard solution (n-hexane solution of 0.5 mg/mL methyl salicylate), and n-hexane was added to make the final volume of 600 μL. A capillary column (numbered BD-WAX122-7032, 30 m × 0.250 mm × 0.25 μm, Agilent Technologies Inc., Santa Clara, CA, USA) was installed after the gas phase instrument had been preheated. The injection procedure was as follows: In Step I, the column temperature was 180 °C; in Step II, the column temperature was increased to 230 °C at a rate of 3.0 °C/min and maintained for 3 min. The inlet temperature was set at 240 °C, and the detector temperature was set at 280 °C [28].

Quantitative determination of yield by internal standard method: 0.5 mg/mL methyl salicylate was used as internal standard to quantitatively detect biodiesel products.

The calculation formula of fatty acid methyl ester conversion rate was as follows [29]:

$$C=\frac{(\Sigma A-A_{IS})\times C_{IS}\times V_{IS}}{A_{IS}\times m}$$

where C represents the conversion rate of fatty acid methyl ester, ∑A and *A_IS_* represent the peak area of all fatty acid methyl esters and the peak area of the internal standard substance methyl salicylate, respectively, *C_IS_* and *V_IS_* represent the concentration (0.5 mg/mL) and volume (mL) of the internal standard substance methyl salicylate, respectively, and m is the mass of soybean oil added (mg).

## Figures and Tables

**Table 1:** SNC input (mg/g carrier), protein immobilization amount (mg/g carrier), protein immobilization efficiency.

SNC Input (mg/15 mL Reaction Solution)	SNC Loading (mg/g Carrier)	Immobilization Yield (%)
0.60	0.59 ± 0.01	99.5 ± 0.8
0.80	0.70 ± 0.01	88.4 ± 1.4
1.00	0.78 ± 0.01	78.4 ± 0.9
2.00	0.80 ± 0.01	40.2 ± 0.6

**Table 2:** The total amount of fusion lipase immobilized (mg/g carrier), 2–12 h protein immobilization (mg/g carrier), protein immobilization efficiency.

PFL Input (mg/g Carrier)	Time (h)	PFL Loading (mg/g Carrier)	Immobilization Efficiency (%)
3.70	2	2.41 ± 0.06	65.1 ± 1.5
3.70	8	3.03 ± 0.01	81.4 ± 0.3
3.70	12	3.25 ± 0.01	87.4 ± 0.4

**Table 3:** Primer information.

Primer Name	Primer Sequences
SNCF	5′-AAACATGCCATGGAACCAACTCCGCCGAC-3′
SNCR	5′-CCGCTCGAGTTATTAGTGGTGGTGGTGATGG-3′
PSNTF	5′-CATGCCATGGATCATCATCATCATCATCATATGTCCCAGGATTC-3′
PSNTR	5′-GGCCTCGAGTTATTATTTGTTAACTTTGATAAATTCGATGTCGC-3′

## Data Availability

The raw data supporting the conclusions of this article will be made available by the authors on request.
